# Harnessing Generalizable Real-World Ophthalmic Big Data: Descriptive Analysis of the Bodhya Eye Consortium Model for Collaborative Research

**DOI:** 10.2196/53370

**Published:** 2024-09-30

**Authors:** Ishaana Sood, Shalinder Sabherwal, Umang Mathur, Elesh Jain, Madhu Bhadauria, Deepshikha Agrawal, Ashi Khurana, Vikas Mittal, Avinash Mahindrakar, Vishal Govindahari, Sucheta Kulkarni, Ken K Nischal

**Affiliations:** 1 Dr Shroff's Charity Eye Hospital New Delhi India; 2 Bodhya Eye Consortium India India; 3 Pediatric Ophthalmology and Strabismus Sadguru Netra Chikitsalaya Chitrakoot India; 4 Regional Institute of Ophthalmology, Sitapur Eye Hospital Sitapur India; 5 MGM Eye Institute Chhatisgarh India; 6 CL Gupta Eye Institute Moradabad India; 7 LJ Eye Institute Ambala India; 8 Pediatric Ophthalmology, Oculoplasty and Neuroophthalmology Srikiran Institute of Ophthalmology Kakinada, Andhra Pradesh India; 9 Pushpagiri Vitreo Retina Institute Hyderabad India; 10 PBMA's HV Desai Eye Hospital Pune India; 11 Division of Pediatric Ophthalmology, Strabismus, and Adult Motility UPMC Children’s Hospital of Pittsburgh Pittsburgh, PA United States

**Keywords:** anthropological and genomic heterogeneity, big data, consortium, collaborative research, generalizability, global health impact, North India

## Abstract

**Background:**

Eye care organizations and professionals worldwide are increasingly focusing on bridging the gap between population health and medical practice. Recent advances in genomics and anthropology have revealed that most Indian groups trace their ancestry to a blend of 2 genetically distinct populations: Ancestral North Indians, who share genetic affinities with Central Asians, Middle Easterners, Caucasians, and Europeans; and Ancestral South Indians, genetically distinct from groups outside the Indian subcontinent. Studies conducted among North Indian populations can therefore offer insights that are potentially applicable to these diverse global populations, underscoring significant implications for global health.

**Objective:**

The Bodhya Eye Consortium is a collaboration among 8 high-volume nonprofit eyecare organizations from across North India. The consortium aims to harness real-world data consistently and with assured quality for collaborative research. This paper outlines the formation of the consortium as a proposed model for controlled collaborative research among the leading eyecare organizations of North India.

**Methods:**

We detail the creation and effective implementation of a consortium following a structured road map that included planning and assessment, establishing an exploratory task force, defining specialty areas, setting objectives and priorities, and conducting a SWOT (strengths, weaknesses, opportunities, and threats) analysis. Central to this process was a comprehensive data audit aimed at standardizing data collection across all participating organizations.

**Results:**

The consortium currently comprises 9 organizations, each represented in the governance structure by the Governing Council. Scientific standards for published research are established and overseen by the Scientific Committee, while the Conflict Resolution Committee manages any unresolved disputes. The consortium’s working groups, organized by various eyecare specialties, collaborate on research projects through virtual interactions. A foundational step in this process was the organizationwide data audit, which revealed that most organizations complied with accurate and standardized data collection practices. Organizations with deficiencies in data completeness developed action plans to address them. Subsequently, the consortium adopted data collection proformas, contributing to the publication of high-quality manuscripts characterized by low dropout rates.

**Conclusions:**

The collaborative research conducted by the Bodhya Eye Consortium—a group of high-volume eyecare organizations primarily from North India—offers a unique opportunity to contribute to scientific knowledge across various domains of eyecare. By leveraging the established heterogeneity of anthropological and genomic origins within the population, the findings can be generalizable, to some extent, to European, Middle Eastern, and European American populations. This access to potentially invaluable, generalizable data has significant global health implications and opens possibilities for broader collaboration. The model outlined in this descriptive paper can serve as a blueprint for other health care organizations looking to develop similar collaborations for research and knowledge sharing.

## Introduction

Eyecare organizations and professionals worldwide are increasingly focused on bridging the gap between public health and medical practice. There has been a recent emphasis on forming alliances to enhance capacity [1], resulting in the rise of public-private partnerships and collaborations between governmental policy makers and high-volume nonprofit eyecare organizations [2].

Central to deriving generalizable knowledge for public health initiatives from any data is understanding the population under study. In India, the majority of nonprofit eyecare organizations conducting clinical and epidemiological research, and establishing baselines for nearly every ocular condition [3-6], are concentrated in the southern region of the country. Although these data are invaluable, their generalisability to other populations outside India may be limited. Recent advances in anthropology and genomics confirm a long-held suspicion: the majority of Indian groups trace their ancestry to a blend of 2 genetically distinct populations. Ancestral North Indians share genetic affinities with Central Asians, Middle Easterners, Caucasians, and Europeans, while ancestral South Indians exhibit genetic ties predominantly within the Indian subcontinent, distinct from groups outside it [7]. In contrast to the relatively homogeneous population of South India, North Indian populations exhibit greater heterogeneity, sharing genetic similarities with Middle Easterners, Central Asians, and Europeans [7], and by extension, with Euro-Americans. As a result, studies conducted on North Indian populations may hold broader applicability to these diverse groups, potentially carrying significant global health implications. This unique genomic diversity found in North India distinguishes it on a global scale. Establishing a network of institutions across North India for collaborative research presents an ideal opportunity to leverage the unique anthropological and genomic diversity of the region to gather substantial real-world ocular data [8]. Real-world data refer to observational data, in contrast to data obtained from randomized controlled trials. Bian et al [9] demonstrated through a systematic scoping review that the quality of real-world data is often inconsistent due to its complex and heterogeneous nature.

The Bodhya Eye Consortium (BEC) is a collaboration among 8 high-volume nonprofit eyecare organizations across North India. This paper describes the formation of the BEC as a proposed model for controlled collaborative research among these leading eyecare organizations. The consortium aims to harness real-world data in a consistent, quality-assured manner.

## Methods

### Planning and Assessment

We used a comprehensive road map consisting of several key components. The idea for the consortium originated from a global eye genetics consortium in which a few organizations such as ours were participants. Therefore, the organizations approached for the BEC were those with similar structures that were already interacting with each other at various common forums, such as the genetics consortium. These organizations had been involved in such settings for at least 10 years before joining the consortium. Ten high-patient-volume organizations from North India were invited to form the consortium, selected based on the location of their main eye hospitals and catchment areas. Initial discussions were conducted on digital platforms, with joint weekly virtual meetings held over a 12-month period.

### Setting Up an Exploratory Task Force

Following these discussions, an exploratory task force was formed to establish shared goals, define the necessary commitments to achieve them, and secure leadership commitment from the institutions. Subsequently, an in-person meeting was held at a mutually convenient eye hospital in Delhi, where the consortium’s scope and structure were finalized. Discussions also centered on the legal framework for the consortium, forming a clear governance structure for clinical and research endeavors, as well as for future project and funding applications.

### Defining Specialty Areas

Within ophthalmology, specialty areas include cataract, cornea, retina, pediatric ophthalmology, glaucoma, oculoplastics, public health, and ocular microbiology. Working groups were established based on these specialties, with each group comprising representatives specializing in the respective fields from each member organization.

### Setting Objectives/Prioritizing

The objectives outlined in the initial discussions were to build research capacity and facilitate knowledge sharing. A consensus was reached to prioritize conducting high-quality research with the goal of publishing scientific papers in top-ranked ophthalmological journals. The selection criteria for these journals were evaluating both the h-index and the median h-index of each MEDLINE-cited ophthalmology journal, along with their published impact factors. Additional objectives were organizing funding for research and ensuring the robustness of data collected and shared among consortium organizations through a comprehensive data audit.

The data audit was conducted across all participating hospitals to verify the accuracy of patient information entered on the face sheet (first page) of the patient files. The audit followed established protocols and was carried out by trained auditors at each center using a standardized proforma. Twenty files from each of the 10 organizations were selected using a predefined methodology to ensure randomization. Each day, a random number was generated, and the patient corresponding to that number accessing services at the hospital had their file selected, over 5 working days per week. This process was repeated over 4 weeks, resulting in a total of 5 patient files selected per week.

Basic patient information such as unique identifier, name, age, gender, contact number, address, and date of examination was recorded. Additionally, the presence of primary and secondary diagnoses, International Classification of Diseases (ICD) coding, procedures or surgeries performed, complications, and consent for procedures were noted. The variables and their coding are detailed in Multimedia Appendix 1. Proportional analysis was conducted and results are presented in percentages.

### Defining Strengths, Weaknesses, Opportunities, and Threats

A SWOT (strengths, weaknesses, opportunities, and threats) analysis of the BEC and its workings was conducted.

### Ethical Considerations

As this is a theoretical/modeling paper that does not use or discuss any patient data, no ethics approval was required. The article adheres to the Declaration of Helsinki.

## Results

### Planning and Assessment

During the initial tele-discussions, 2 organizations dropped out, citing their inability to sustain contributions. However, approximately 4 years later, 1 of these organizations rejoined the consortium after establishing their catchment area and demonstrating the ability to provide sustained input. In recent years, additional organizations from across India—not just North India—have been included in the consortium. These new additions differ from existing members in terms of research capacity, geographic location, or community experience. This diversity enhances the consortium’s research validity by extending its applicability to populations beyond North India.

### Setting Up an Exploratory Task Force

**Figure 1:**
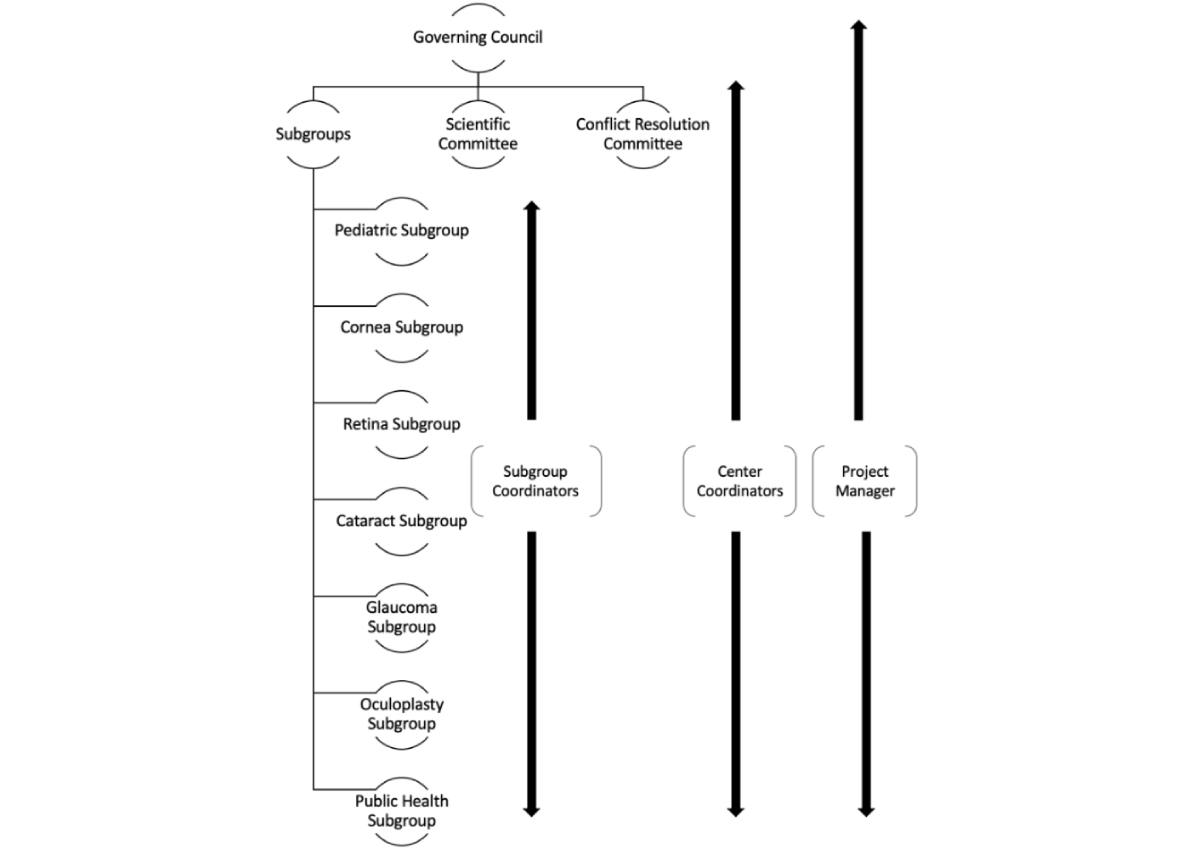
Governance structure of the Bodhya Eye Consortium (BEC).

The governance structure of the BEC was established (Figure 1). At each level of governance, all organizations are equally represented, ensuring similar roles across all members. The Governing Council comprises the Heads of Institutions from each member organization of the BEC. The Scientific Committee, which sets research standards and guidelines for ethics and authorship in collaboration with the Governing Council, includes 2 appropriately trained members from each organization. These members are nominated by the head of each institution and possess a keen interest and experience in research. The Scientific Committee also includes members from diverse backgrounds, including clinical specialists from various subspecialties, public health experts, and basic scientists, including those specializing in genetics. This diversity ensures that research topics remain relevant and innovative. The Scientific Committee is overseen by the Lead Coordinator (a nonvoting member). Additionally, a BEC Project Manager monitors project timelines and ensures ongoing progress, reporting directly to the Governing Council. The Scientific Committee and Governing Council play crucial roles in upholding research standards and quality assurance, serving as the primary decision-making and regulatory bodies of the BEC. Consequently, their responsibilities include ensuring compliance with all legal requirements. Members of both committees hold senior positions within their respective organizations, making it integral to their daily operations to uphold these standards.

To address potential conflicts related to intellectual property and data sharing among multiple organizations, and to ensure appropriate credit allocation, particularly regarding authorship, a Conflict Management Committee was established. This committee reports to the Governing Council and is chaired by an external member of the consortium, who is not affiliated with any of the collaborating organizations within the consortium. The primary role of this committee is to arbitrate disputes within subgroups that cannot be resolved through discussion, particularly regarding authorship concerns. Additionally, the presence and structure of institutional review boards and ethics committees were confirmed across all organizations.

### Defining Specialty Areas

The specialty-focused working groups serve as virtual forums where members discuss, develop, and conduct research. Research topic selection is determined at the working group level, comprising specialists in the respective fields.

Typically, a member of an organization pitches a research topic to the group along with a brief write-up. This proposal is circulated among subgroup members who review it, suggest modifications, and assess their centers’ capacity to participate. Once the participating centers are finalized, they create a data collection proforma and initiate institutional review board approval processes at their respective centers before beginning data collection.

The overall standardization and quality of research topics within the consortium are ensured by the Scientific Committee through thorough reviews of study proposals. The study is spearheaded by the member or center proposing the research, and once the proposal is finalized and the lead center obtains institutional review board approval, the study lead submits the proposal to the Scientific Committee for review. After receiving feedback from the Scientific Committee, the study lead revises their proposal accordingly and resubmits it for further review. Once approved by the Scientific Committee, the data collection process commences.

This process enables each subgroup to conduct multiple studies simultaneously, allowing different centers flexibility in determining their contributions and enabling various members to lead and complete their own studies. The Scientific Committee’s establishment of research processes and authorship guidelines reinforces this approach. Additionally, the Scientific Committee reviews each study’s final manuscript before submission, assessing its scientific relevance, the added value of multicenter involvement, and how these aspects are presented in the manuscript.

Each working group is led by conveners chosen from within the group, who rotate every 3 months to oversee adherence to research guidelines and task deadlines. Administrative support for the working groups is provided by coordinators, 1 from each center, responsible for recording meeting minutes, tracking tasks, and coordinating with other consortium centers and stakeholders. Quarterly updates from conveners to all consortium members regarding subgroup progress encourage the cross-pollination of ideas.

Virtual monthly interactions foster cooperation, collaboration, and mutual respect among all participants through real-time discussions and debates. These interactions also facilitate knowledge exchange and capacity building within member organizations, exemplified by the establishment of virtual grand rounds and research workshops. Virtual grand rounds serve as a monthly knowledge-sharing platform open to all clinical personnel from member organizations, conducted on a rotating basis across each specialty area. Research workshops are conducted to foster a research culture, providing training to staff on research fundamentals such as formulating research questions, conducting literature reviews, interpreting statistical analyses, and reading journal articles. These workshops are open to all clinical and research staff of consortium hospitals, encouraging both in-person and virtual discussions. Workshops are held at member organizations on a rotating basis. One impactful example is the microbiology training, where 2-3 paramedical personnel from each partner institution attended a 6-day training at the microbiology department of one of the founding consortium members. The training included theoretical materials and starter kits with stains and slides, aimed at establishing basic microbiology services to enhance keratitis diagnosis at their hospitals and to set up a network for future research projects.

### Setting Objectives/Prioritizing

One of the primary activities aimed at ensuring data robustness was a file audit, conducted to standardize routine data collection processes across member organizations before formally initiating research and data sharing within the consortium. A total of 200 randomly selected files were audited across all 10 participating organizations at the time.

All 200 files (100%) included the patient’s name, age, gender, contact number, address, and date of examination. Of these, 130 files (65%) reported both primary and specialty diagnoses, while 50 files (25%) reported only the primary diagnosis, with the specialty diagnosis available in detailed clinical records but missing from the diagnosis field; 2 files (1%) had incomplete diagnoses with the critical information entered, 1 file (0.50%) had incomplete diagnosis with critical information missing, and 7 files (3.5%) had no diagnosis entered. Among the 200 files audited, 162 (81%) files had complete and accurate ICD coding. Additionally, 10 (5%) files had complete but inaccurate coding, and 8 (4%) files had incomplete coding. Furthermore, 22 (11%) files did not have any ICD coding at all. Regarding procedure or surgery reporting, 158 (79%) files fully documented the procedure or surgery undergone by the patient, including the date. In 4 (2%) files, the procedure or surgery was noted without the date, and in 2 (1%) files, it was incomplete. Moreover, 38 (19%) files did not have the procedure or surgery entered at all. Concerning patient consent, 184 (92%) files indicated that patients had consented to undergo the procedure or surgery, while 16 (8%) files did not document patient consent.

All organizations participating in the BEC collect and store data using electronic medical records. Fields with more than 10% missing data were identified and highlighted to improve practices for clinical data–based studies. Each organization identified its deficiencies, developed action plans to address them, and implemented solutions. Future consortium members undergo the same data audit process upon joining.

This initiative subsequently introduced the use of proformas to facilitate the recording of reproducible and accessible data within working groups. For instance, in pediatrics, proformas were utilized to capture not only a patient’s vision data but also the method of acquisition. Additionally, proformas were used to gather information from patient notes, and a feasibility form was circulated before proposing studies to assess the clinical and research capabilities at each center. Based on this approach, each center assessed its ability and capacity to participate in studies proposed through the consortium’s subgroups. As a result, not all centers participate in every study. This strategy enabled us to plan, execute, and publish our first study [10], which involved participation from 3 of the consortium centers. While some variations are natural in clinical practice, we ensure that all planned research maintains uniform diagnostic and treatment practices across participating centers. Currently, data heterogeneity is addressed at the outset of each research endeavor, which may result in excluding centers unable to meet required standards.

With the objective of publishing a high-quality manuscript, the first study conducted under the consortium describes the clinical features, visual acuity, and causes of ocular morbidity in 532 children (0-18 years) from North India with microphthalmos, anophthalmos, and coloboma. This study has been published in a major international journal [10]; however, 3.2% (17/532) of the data needed to be excluded as a result of quality issues. Since then, collaborative efforts have successfully completed various research projects based on both retrospective and prospectively collected data. A total of 8 peer-reviewed studies have been published (n=6) or accepted (n=2) in international and national journals [11-16]. Currently, the consortium’s research effectiveness is gauged by its capacity to consistently publish high-quality research.

### Define Strengths, Weaknesses, Opportunities, and Threats

The SWOT analysis is detailed in Table 1.

**Table 1:** Strengths, weaknesses, opportunities, and threats of the Bodhya Eye Consortium.

Strengths	Weaknesses	Opportunities	Threats
Abundance of talented providers and scientists within member hospitals. High-volume clinics allowing access to big data. Determination to establish a strong pedigree of research and clinical excellence. Extraction of quality retrospective data.	Time as a limiting factor in terms of planning and conducting organized research. Stresses that come with working in regular eyecare service delivery organizations. Lack of a central monetary source. Lack of a data-sharing agreement.	Funding for research and administration.	Dependence on social media platforms and security of conducting regular communications and sharing medical data.

## Discussion

### Principal Findings

The BEC was established through a formal collaboration among existing high-volume clinical eye centers in North India. There is limited literature on the formation of such collaborative processes, particularly in eyecare [17]. The BEC was founded using a standardized approach that included developing organizational structures; standardizing data collection systems [18]; establishing research protocols and guidelines; training personnel appropriately; and using advanced tools, techniques, and technology to enhance operational efficiency and knowledge advancement. The value of such a consortium lies in its diverse geographic locations and strong interinstitutional collaboration.

As a diverse country representing populations of varying demographics, India offers a rich pool for genetic analysis with the potential to deliver significant global impact [8]. The unique geographical spread of the BEC enables studies with globally generalizable data (excluding Africa) [8], leveraging the large patient numbers and high-quality big data collected through this collaboration of nonprofit eyecare organizations. This plays a significant role in advocacy and has the potential to influence government priorities in allocating funds for research and disease control, particularly in eyecare and specific conditions.

The collaboration has been facilitated through the use of digital platforms, which streamlined communication and enabled the BEC to maintain operations during the COVID-19 pandemic and subsequent lockdown [19]. Collaborative multicenter research aims to enhance patient care through improved data quality [20], driven by the commitment and engagement of clinical researchers dedicated to translational work.

Grand rounds and workshops have improved skill and knowledge levels, fostering a better understanding of research methodology. Once the consortium’s bank account is established, pooled funds will enable us to promote these events on our website and open them to the broader public based on demand. This progressive approach has already enabled collaboration with organizations such as the Global Eye Genetics Consortium [21], facilitating access to resources such as guest speakers and subject experts from around the world—opening new avenues of thought and fostering network development.

The Scientific Committee is also actively developing advanced policies for authorship, data sharing, and research processes. Increasing in-person meetings and collaborative sessions would further enhance interdisciplinary collaboration among member organizations of the consortium. Member organizations already include scientists, geneticists, public health specialists, and microbiologists as part of their representation in subgroups, complementing clinicians. This diversity enables the consortium to explore new interdisciplinary studies, leveraging different perspectives.

To ensure proficiency in research methodologies and data management among all personnel, the Scientific Committee conducts regular workshops to enhance these skills. Plans are underway for a series of continuous online workshops and activities for ongoing skill development. Discussions are also ongoing to mandate that principal investigators undergo training and certification from selected institutions before their study proposals are approved by the Scientific Committee.

As a foundational activity, the data audit has proven especially beneficial for ensuring the quality and accuracy of data during collection, addressing persisting drawbacks even in retrospective studies [22,23]. Once the consortium establishes a joint bank account and begins sharing finances, it plans to hire a traveling manager. This manager will travel across organizations to ensure standardization in data collection practices, quality, storage, and consent procedures. This initiative will enhance the consortium’s data collection methods, ensuring better standardization and consistent quality across all member organizations [24]. Currently, the analysis for consortium studies is conducted by a biostatistician from 1 of the participating centers, dedicating approximately 20% of their time to consortium studies, including those not led directly by their center. Similar to the traveling manager, there are plans to hire a dedicated biostatistician for the BEC in the long run.

Upon acceptance of the first publication under the BEC’s auspices [10], editors emphasized that the paper’s key strengths included its high-quality multicentric data and the inclusion of a large number of patients within a short time frame. Furthermore, the use of proforma-driven data recorded during consultations ensured a low patient exclusion rate (17/532, 3.2%) in this retrospective study, enhancing its value in the field and minimizing the need for techniques to address data “missingness” [25]. Currently, there are nearly 30 ongoing studies at various stages of the writing and submission process, with 6 already published [11-16] and 2 more accepted. While the consortium is currently in a nascent stage and its research impact is primarily measured through publications, in the medium to long term, as we become more influential in health policy and behavior change, we will broaden our evaluation criteria to include not only impact factors but also other relevant indicators. Furthermore, as we establish a mechanism to create a shared pool of resources, we aim to publish in higher impact factor open-access journals, many of which require publication fees. This initiative will enable us to monitor impact factors and citation counts as quality indicators for journal reach, prestige, and impact. Currently, all current members of the BEC have undergone standardization audits, and scaling up will inevitably introduce additional variables into consideration.

Although initial efforts have begun with retrospective “data-only” studies and a limited number of prospective studies, future endeavors involving extensive prospective studies and biological sample collection will increase research costs, necessitating groundwork in terms of protocols and monitoring activities. While there is encouragement to initiate large prospective studies and collect biological samples, the consortium requires an expansive and reliable framework to streamline public health and clinical studies before embarking on larger studies involving biological tissue collection.

As consortium research intensifies, strategies can be implemented to address time constraints faced by researchers and clinicians. These strategies may include incentivizing participation in research by allocating dedicated time to senior faculty, as well as providing administrative support to ensure clinicians can focus solely on research activities. Additionally, the consortium’s current community engagement is primarily through research endeavors, such as a study to assess the awareness, knowledge, and challenges faced by beneficiaries and nonbeneficiaries of Ayushman Bharat—Pradhan Mantri Jan Arogya Yojana, which has been accepted for publication in a renowned peer-reviewed journal. However, as we conduct more studies actively engaging with local communities, such as needs assessments and qualitative studies, we aim to gather evidence to strengthen the impact and relevance of our work. Therefore, more of these studies are being initiated within the BEC. An example of this is our Glaucoma Subgroup, which is studying awareness of glaucoma, and our Public Health Subgroup, which is investigating awareness of eye health among rural women. As we conduct more studies like these, we plan to utilize various platforms to disseminate our findings and use the evidence for advocacy purposes.

Moreover, in today’s dynamic environment, with social media platforms frequently updating their privacy and encryption policies, the sharing of anonymized medical data raises significant concerns. However, we maintain strict monitoring of communications, ensuring that patient data are never discussed over social media. Despite this, given the evolving landscape of social media privacy policies and the introduction of the new Digital Personal Data Protection Act of 2023 [23], there is an urgent need for a more robust and secure data-sharing platform. The new act allows for the legitimate use of medical data when patients voluntarily enroll themselves [26]. However, because research is exempt from legitimate use under the act, updating patient consent forms to include data sharing for collaborative consortium research among member organizations becomes crucial to ensure compliance. In the medium to long term, the BEC can address the necessity for a secure data-sharing and communication platform. Working with relevant professionals, it can develop a digital infrastructure tailored to its specific requirements. Our aspiration is to establish a dedicated platform for medical and health research, ensuring encrypted communication and strict adherence to privacy regulations for sharing medical data. A member organization is currently testing such a platform at an individual level, and based on their feedback, we plan to integrate it into consortiumwide use. These resources are critical for maintaining data validity and quality in research, and for enhancing the impact of prospective studies.

The initial step toward securing sustainable funding for ongoing and future research projects involves establishing a formal data-sharing agreement and subsequently opening a joint bank account to facilitate shared financial management. This process has already begun and will enable the consortium to pursue collaborative applications for national and international research grants. Additionally, we plan to approach funders already associated with these organizations for service delivery. The programmatic sustainability of the consortium is currently ensured through regular meetings of its subgroups and other bodies, quarterly meetings, and established monitoring and motivating mechanisms. Financial sustainability will become crucial in the medium to long term once the consortium opens a joint bank account and establishes a Finance Committee. Therefore, establishing appropriate financial mechanisms, including processes for collective fund contributions, fundraising for grants, and their disbursement, will be a priority. Additionally, funding efforts will be bolstered by publishing a descriptive methodology paper to position the consortium in scientific forums.

Currently, the Heads of Institutions are working to finalize and sign a comprehensive legal and financial agreement. With the Indian Ministry of Law and Justice introducing an Act to govern and regulate health care data [26], it is imperative to expedite this process in accordance with the new regulations. We continuously update our practices in line with the latest Data Protection Act and its modifications. Some organizations are already conducting regular Good Clinical Practice training for all researchers. We plan to extend this training to all member organizations once we have the necessary resources. Until a uniform data-sharing agreement can be adopted, each research endeavor collects only anonymized data through proformas to facilitate ease of operation at the subgroup level. Each institution obtains separate approvals from its respective institutional review boards and ethics committees—a time-consuming process from start to finish [27]. Furthermore, the study lead must also apply for and obtain approval from the Scientific Committee, which evaluates the proposal’s relevance as a multicentric study and the consortium’s role within it. To streamline this process, we are working to credential Scientific Committee members as representatives of each member organization with institutional review boards. We also aim to include external members from the United Kingdom and the United States. This approach will allow principal investigators of consortium studies to receive uniform central-level approval and detailed feedback before proceeding with individual organization-level applications.

In the short to long term, adopting sound financial practices and implementing well-structured, transparent systems, coupled with significant publications such as those already secured, could attract global research grants. This approach would also enable the consortium to establish a financial reserve to support its activities and implement secure, encrypted data-sharing structures. The consortium can also focus on expanding its reach and impact on global health beyond its current genomic and anthropological focus. Given its diverse group of collaborators, including basic scientists, clinicians, public health researchers, and epidemiologists, the consortium is well-positioned for this expansion. Consortiumwide collaborations with existing partners at the International Centre for Eye Health, the London School of Hygiene and Tropical Medicine, the University of Wisconsin–Madison, the University of Iowa, and the University of Pittsburgh can also be planned and executed. These are the administrative objectives of the consortium.

The consortium also has future scientific objectives aligned with the global health research agenda [28,29], particularly focusing on priorities set by the World Health Organization and the International Agency for the Prevention of Blindness, such as effective cataract surgical coverage, refractive error, and myopia. Collaborative studies are already underway on these topics across various subgroups. Long-term goals include advancing into advocacy and policy influence through high-quality, high-volume research.

### Conclusions

Our consortium offers a distinctive opportunity for members to advance scientific knowledge across various domains of eyecare. The population diversity in North India, encompassing heterogeneity in anthropological and genomic origins, allows findings from our studies to be somewhat generalizable to European, Middle Eastern, and European American populations. The consortium holds significant global health implications, and the model outlined in this descriptive paper could serve as a blueprint for other health care organizations seeking to establish similar collaborations for research and knowledge sharing.

## Appendix

Multimedia Appendix 1 Variables and their coding for the data robustness analysis.

## Abbreviations

BEC: Bodhya Eye Consortium
ICD: International Classification of Diseases
SWOT: strengths, weaknesses, opportunities, and threats
