# Supplementary material for: Harnessing Generalizable Real-World Ophthalmic Big Data: Descriptive Analysis of the Bodhya Eye Consortium Model for Collaborative Research
Source: Online J Public Health Inform. 2024 Sep 30;16:e53370. doi: 10.2196/53370 (PMC11474137; doi:10.2196/53370)
Supplement: Multimedia Appendix 1 [file ojphi_v16i1e53370_app1.docx]

| **Variable** | **Coding Based on Presence on Facesheet** | | | | |
| --- | --- | --- | --- | --- | --- |
| Name | Yes | No |  | | |
| Age | Yes | No |  | | |
| Gender | Yes | No |  | | |
| Contact Number | Yes | No |  | | |
| Address | Yes | No |  | | |
| Date of Examination | Yes | No |  | | |
| Primary and Secondary Diagnosis on Facesheet | 0: None entered | 1: Primary diagnosis incomplete or missing for one eye and important diagnosis not entered | 2: Primary diagnosis incomplete or missing for one eye but important diagnosis entered | 3: Primary diagnosis complete but speciality absent (where applicable) | 4: Primary and speciality diagnosis complete |
| ICD Coding | 0: None entered | 1: Incomplete (part of diagnosis missed) | 2: Complete but inaccurate | 3: Complete and accurate |  |
| Procedure/Surgery | 0: None entered | 1: Entered but incomplete | 2: Complete but without date | 3: Complete with date |  |
| Complications | 0: Not entered | 1: Entered | 2: Not applicable |  | |
| Consent for Procedures | 0: Absent | 1: Present | 2: Not applicable |  | |
